# Stroke risk evaluation for patients with atrial fibrillation: Insights from left atrial appendage

**DOI:** 10.3389/fcvm.2022.968630

**Published:** 2022-08-22

**Authors:** Runxin Fang, Yang Li, Jun Wang, Zidun Wang, John Allen, Chi Keong Ching, Liang Zhong, Zhiyong Li

**Affiliations:** ^1^School of Biological Science and Medical Engineering, Southeast University, Nanjing, China; ^2^Zhongda Hospital, The Affiliated Hospital of Southeast University, Nanjing, China; ^3^First Affiliated Hospital, Nanjing Medical University, Nanjing, China; ^4^Duke-NUS Medical School, National University of Singapore, Singapore, Singapore; ^5^National Heart Centre Singapore, Singapore, Singapore; ^6^School of Mechanical, Medical and Process Engineering, Queensland University of Technology (QUT), Brisbane, QLD, Australia

**Keywords:** left atrial appendage, atrial fibrillation, stroke, association, risk evaluation

## Abstract

Left atrial appendage (LAA) is believed to be a common site of thrombus formation in patients with atrial fibrillation (AF). However, the commonly-applied stroke risk stratification model (such as. CHA_2_DS_2_-VASc score) does not include any structural or hemodynamic features of LAA. Recent studies have suggested that it is important to incorporate LAA geometrical and hemodynamic features to evaluate the risk of thrombus formation in LAA, which may better delineate the AF patients for anticoagulant administration and prevent strokes. This review focuses on the LAA-related factors that may be associated with thrombus formation and cardioembolic events.

## Introduction

Atrial fibrillation (AF) is the most common sustained rhythm disorder worldwide (1–4), and the worldwide AF incidence is approximately 1–2% (3–6), and the prevalence increases with age. In general, patients with AF have higher morbidity and mortality rates due to increased risk of stroke (7–10). The risk of ischemic stroke related to AF, increases from 4.6% for ages 50 through 59 years to 20.2% for ages 80 through 89 years (11). Therefore, AF prevalence is expected to dramatically increase with an aging population in the coming years (12).

In AF patients, irregular heart rhythm disturbs blood flow, which can lead to blood stagnation and clot formation, with subsequent dislodgment and embolization in the brain resulting in thromboembolic events (13–15). There is an agreement that AF increases stroke risk by 5-fold (16), where AF-related strokes are associated with greater disability, higher medical cost, poorer functional outcomes, and a lower chance of being discharged to home compared to non-AF-related strokes (17).

In AF patients with thromboembolic event, studies have revealed that more than 90% of thrombi originate in the left atrial appendage (LAA) (18–20), which is the remnant of the embryonic left atrium and originates from the primordial pulmonary vein and its branches (21–24). However, the commonly applied stroke risk scoring model (*viz*.: CHA_2_DS_2_ or CHA_2_DS_2_-VASc, shown in the following Table 1) in the clinic incorporates only patient demographics and clinical data (25), but do not include any morphological or hemodynamic information about the LAA, and we think that with the LAA information added, some high stroke risk patients but with low scores would be identified.

**Table 1:** Definition of CHA_2_DS_2_-VASc or CHADS_2_ (17).

**Letter**	**Risk factor**	**Score**
C	Congestive heart failure	1
H	Hypertension	1
A_2_	Age≥75	2
D	Diabetes mellitus	1
S_2_	Stroke history	2
V	Vascular disease	1
A	Age 65–74	1
Sc	Sex category (i.e., female sex)	1

Recent studies have shown extensive evidence that it is necessary to include some important LAA features to better evaluate the thromboembolic risk and prevent stroke risk. This study serves to overview of current concepts and recent developments in the relationship between stroke risk and LAA risk-related characteristics.

## LAA characteristics related with stroke

A number of studies have focused on the stroke-related LAA characteristics. These characteristics can be classified into three groups: shape, morphology and hemodynamics. In the following paragraphs we elucidate the relationship between these characteristic groups and stroke risk.

### LAA shape

**Figure 1:**
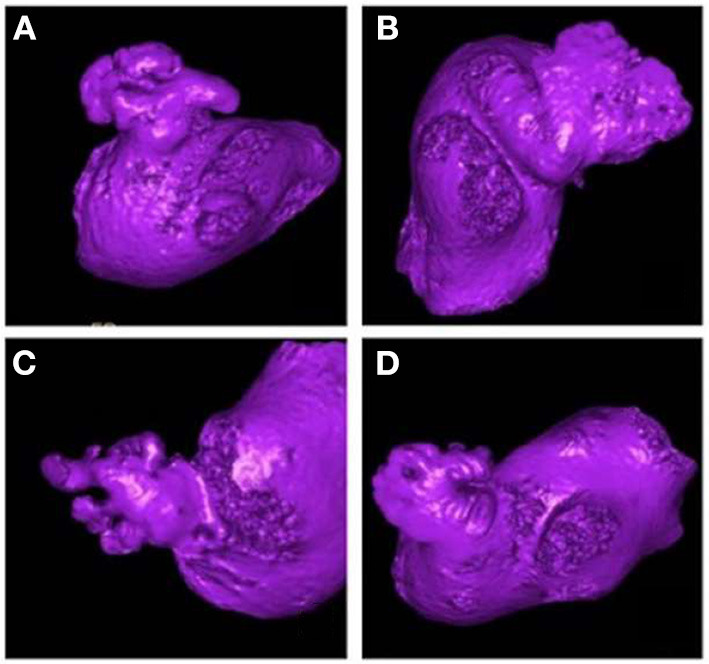
The shape classification of LAA. **(A)** Cactus, **(B)** Windsock, **(C)** Cauliflower, **(D)** Chicken wing. This picture is driven from the classification performed by the clinicians based on Wang's scheme (30).

Shape is an intuitive term for describing the external form, contours, or outline of the LAA, and various classification approaches have been proposed for describing LAA shape (26–29). In these classification schemes, a widely accepted classification, first proposed by Wang et al. (26), classifies LAA into 4 types: cactus, chicken wing, windsock and cauliflower (Figure 1). This classification was derived from 612 patients based on characteristics of bend, lobes and overall length. Where the characteristics were used as the guidance for LAA closure.

Di Biase et al. (31) conducted an initial study to investigate the relationship between LAA shape and stroke risk. In their study, 932 AF patients were classified into four groups based on Wang's shape types obtained using computer tomography (CT)/magnetic resonance imaging (MRI), and they found that the chicken wing LAA type was less likely to have a stroke/transient ischemic attack (TIA), and the cauliflower was the highest stroke risk type. As the first study to focus on the association between stroke risk and LAA shape, Di Biase's research obtained considerable attention. Subsequent independent investigations conclusively established chicken wing as the “safe” LAA type (32, 33), and from that point to present, the consensus that chicken wing type LAA is least likely to result in stroke (i.e., is the “safe” LAA type) is widely accepted in clinical practice (34).

After recognition of chicken wing as the LAA shape with lowest risk, questions remained regarding high-risk LAA shapes, motivating a series of investigations to figure out the “unsafe” LAA types, which found that all the three non-chicken wing LAA shapes were “unsafe” (35–39). Specifically speaking, Lee et al. (39) conducted a case-control study on 255 patients based on the history of cardiovascular adverse events, and concluded that the cauliflower type is associated with an increased risk of stroke. Deng et al. (37) evaluated stroke risk *via* detection of spontaneous echo contrast (SEC) or thrombi using 3D transesophageal echocardiography (TOE) in 320 AF patients and found that the cauliflower LAA type was associated with a higher prevalence of SEC or thrombi. Adukauskaite et al. (35) found that windsock was the highest stroke-prone LAA type based on a stroke history assessment of 158 patients. Anselmino et al. (36) reached the conclusion that cauliflower and windsock are associated with a higher prevalence of silent cerebral ischemia based on a study of 348 AF patients. Kimura et al. (38) performed a case-control study investigating the high-risk LAA type in 80 AF patients with low CHADS_2_ score, and found cauliflower to be the most stroke-prone LAA type. Additionally, Smit et al. (40) developed a new 4-type classification scheme (chicken wing, swan, cauliflower and windsock) and evaluated stroke risk as a function of prior stroke history. Their results demonstrated that the swan LAA type (LAA presented a second sharp curve folding the structure back) was independently associated with the prior stroke history. Given the results of these studies, researchers subsequently combined the three “non-chicken wing” shapes into a single category and conducted studies to confirm an “unsafe” designation for risk of stroke on the “non-chicken wing” type (41–43). The simplification and efficiency of this two-type classification scheme is widely recognized in clinic (33, 34).

However, it is worth mentioning that several studies did not justify the origin of the emboli, as many studies considered that most strokes for AF patients are resulted from the cardioembolic origin. We think it is important to determine the embolic origin in future studies.

Detailed information on Wang's scheme of LAA shape types and the related studies on the association between stroke risk is summarized in Table 2.

**Table 2:** Studies on association between stroke risk and LAA shape based on Wang's scheme.

**LAA classification**	**References**	**Patients**	**Imaging modality**	**Judge for stroke risk**	**Conclusions**	
**Wang's model**: cactus, chicken wing, windsock and cauliflower	Di Biase et al. (31)	932	CT/MRI	Prior stroke	**Chicken wing** less likely to have suffered the prior stroke; **Cauliflower** more likely to have suffered a cerebrovascular ischemic event	“Safe” LAA type
	Lee et al. (32)	360	CT	Prior stroke	**Chicken wing** associated with lower prevalence of stroke	
	Luperico et al. (33)	2596	CT/TEE/MRI	Prior thromboembolic events (TE)	**Chicken wing** less likely to develop TE	
	— — — — — — — — — — — — — — — — — — — — — — — — — — — — — — — — — — — — — — — — — — — — —					
	Lee et al. (39)	255	CT	Prior CETIA or CES	**Cauliflower** associated with increased risk of CETIA and CES	“Unsafe” LAA type
	Adukauskaite et al. (35)	158	CT	Prior cardio-embolic (CE) stroke	**Windsock** associated with CE stroke	
	Deng et al. (37)	320	TEE	Detection of SEC or thrombi	**Cauliflower** associated with higher prevalence of SEC or thrombi	
	Anselmino et al. (36)	348	CT/MRI	Silent cerebral ischemia (SCI) burden	**Cauliflower** and **windsock** associated with prevalence of SCI	
	Kimura et al. (38)	80	CT	Prior stroke	**Cauliflower** considered as the most stroke-prone LAA type	
	Smit et al. (40)	908	CT	Prior stroke and/or transient ischemic attack	**Swan** shape independently associated with prior stroke/TIA	
— — — — — — — — — — — — — — — — — — — — — — — — — — — — — — — — — — — — — — —— — — — — — — — — — — — — — — — —						
**Two types**: chicken wing &non-chicken wing	Kong et al. (41)	219	CT	Prior stroke	**Non-chicken wing**: an independent predicator of stroke	
	Du et al. (42)	555	CT	Detection of LA/LAA thrombus	**Non-chicken wing**: a powerful predicator of LA/LAA thrombosis	
	Yaghi et al. (43)	172	CT	Prior stroke	The prevalence of **non-chicken wing** type higher in patients with CE stroke	

TEE, Transoesophageal echocardiography; MRI, Magnetic Resonance Imaging; LAA: left atrial appendage.

Although Wang's scheme is widely accepted in clinical application, however, several studies have revealed marked variability in shape classification based on the scheme. Both Wu et al. (44) and Khurram et al. (45) pointed out the high inter-observer variability in the classification schemes based on Wang's shape description.

Table 3 illustrates the diversity in the prevalence among Wang LAA shape types observed by different investigators. Such disparity indicates that rigorous ascertainments based on these rather subjective descriptors is difficult and not reliably reproducible. The need for greater objectivity in defining LAA classification parameters enabling reliable assessment of associations with stroke risk led to the development of morphology parameters.

**Table 3:** Prevalence of LAA shapes in different studies.

**References**	**Sample size**	**Prevalence (%)**			
		**Chicken wing**	**Cactus**	**Windsock**	**Cauliflower**
Wang et al. (26)	612	18.3	5.9	46.7	29.1
Di Biase et al. (31)	932	48	30	19	3
Lee et al. (32)	360	43.1	30	13	13.9
Deng et al. (37)	320	14.1	42.8	13.4	29.7
Du et al. (42)	555	67.9	18.7	11.2	2.2

### LAA morphology

The LAA morphology classification approach, with characteristics categorized by means of dimensional level, is superior to the LAA shape scheme in terms of objectivity and reproducibility and is widely studied.

**Figure 2:**
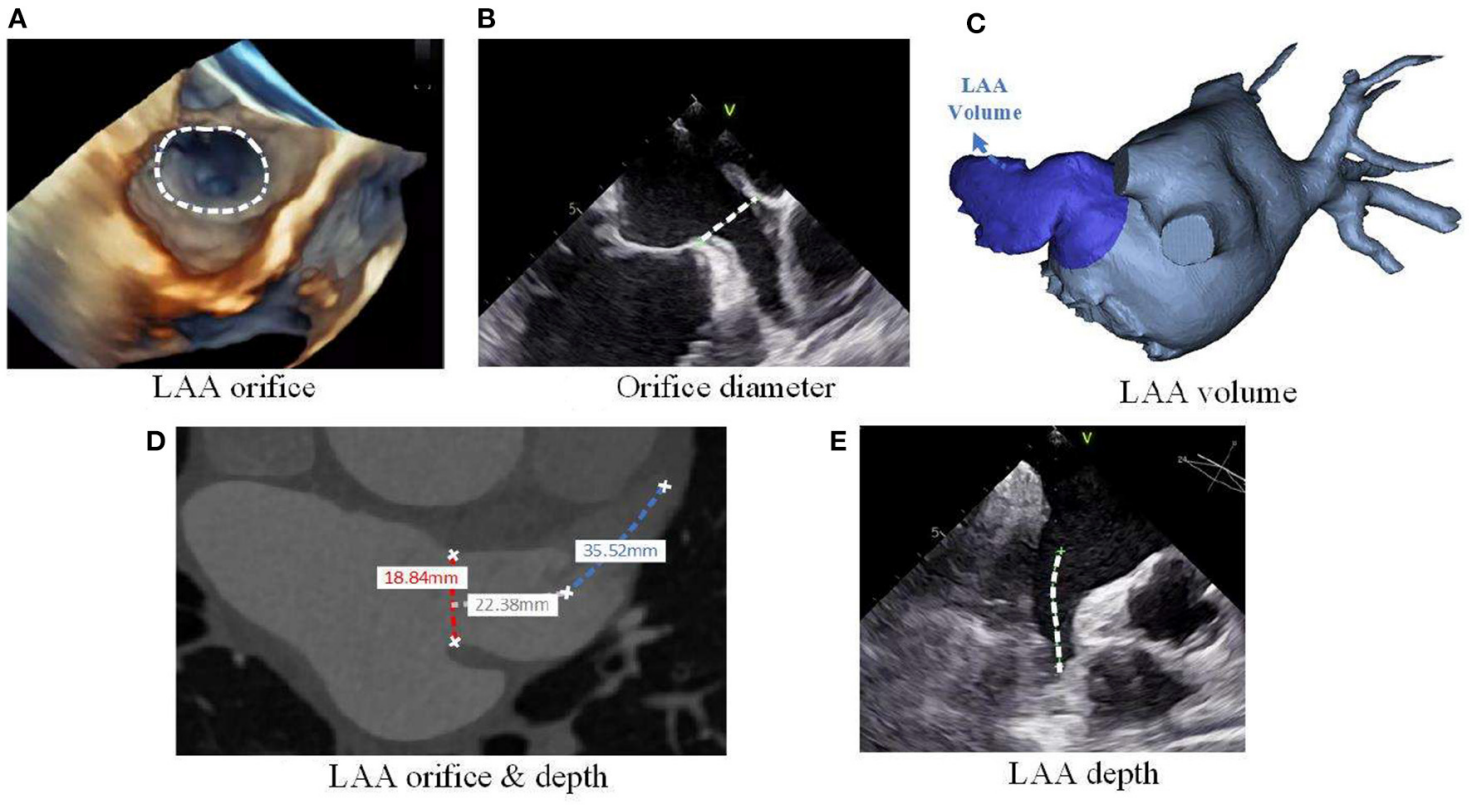
LAA morphological parameters. **(A)** LAA orifice measurement from 3D echo, **(B)** LAA orifice diameter measurement from planer echo, **(C)** LAA volume measurement from 3D reconstruction of CT images, **(D)** LAA orifice & depth measurement from planer CT images, and **(E)** LAA depth measurement from planar echocardiogram.

Among these morphological characteristics, the LAA orifice (see Figure 2A) attracts a lot of focus, and a number of studies have revealed the correlation between orifice size and the stroke risk. In these studies, LAA orifice is consistently shown to be positively related to the stroke risk, both for its area (35, 46, 47) and diameter (41, 45, 48–50) (see Figure 2B). Although the positive relationship is widely accepted, the cut-off value of the orifice size for determining the high stroke risk is seldom calculated. Only Lee et al. (46, 47) gave the different criteria of >3.5 cm^2^ and >4 cm^2^ respectively in their two studies, with the criterion being larger for those with low CHA_2_DS_2_-VASc scores.

**Figure 3:**
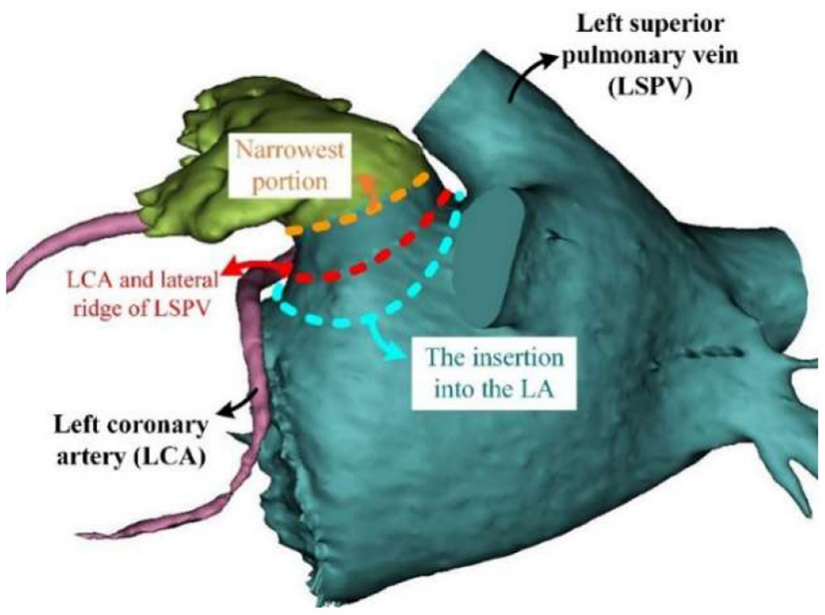
Different definition of LAA orifice. LA, left atrial; LAA, left atrial appendage; LCA, left coronary artery; LSPV, left superior pulmonary vein.

Though the large orifice is believed to relate to the high stroke risk, Khurram et al. (45) draw a contrary conclusion that a smaller LAA orifice diameter is associated with prevalent stroke. In seeking for the reasons for such different conclusions regarding the association between LAA orifice and stroke risk, we found that these studies used different definitions of LAA orifice (Figure 3).

Intuitively speaking, the LAA orifice would be the section at the interface between the LAA and LA (Figure 3, blue dashed line). This intuitive definition was used by Khurram et al. (45). However, an anatomical definition of the LAA orifice exists, and it is usually specified as the section aligning with the left upper pulmonary vein ridge and the circumflex coronary (51) (Figure 3, red dashed line). This seems more accurate and less subjective compared to the intuitive definition, and is used as the measurement method for LAA orifice in (52). However, several studies have defined the LAA orifice as the narrowest portion at the entrance into the left atrium (49, 53). We deduced that this wide-spread definition had its origins in studies of LAA occlusion, as the location of occlusion is usually defined as the narrowest portion (Figure 3, orange dashed line), which would be defined as the LAA neck in our context. The differing definitions caused the diversity of conclusions. In this regard, a clear and unified definition of LAA orifice would be required in the future studies.

Apart from LAA orifice, LAA volume (see Figure 2C) is another prominent parameter. Several studies on the associations between LAA volume and stroke risk have shown that AF patients with a history of stroke/TIA tend to exhibit a larger LAA volume (48, 49, 54–57). However, the specific cardiac phase for defining the LAA volume was not clearly defined in most of the above-mentioned studies. Only Chen et al. (55) clearly stated their LAA volume was at the end-diastolic phase, and the results showed that LAA end-diastolic volume was positively associated with the stroke risk. Although consensus has been achieved regarding a qualitative relationship between the LAA volume and stroke risk, a cut-off value or quantitative relationship was not defined in most of the related studies. Chen et al. (55) and Burrell et al. (54) proposed cut-off values of 8.6 and 34 cm^3^, respectively, for judging stroke risk, although the two values reflect a large disparity which could be attributed to differences in cardiac cycle phase. In this regard, the specific cardiac cycle phase of LAA volume should be defined clearly in future studies, which will allow a standardized comparison among different studies. However, it is worth to mention that the LA will be dilated with the impact form AF, and so the volume of LAA will also be enlarged. Meanwhile, these morphology characteristics would be better compared with the normalizations to the other patient specific characteristics [e.g.,: body mass index (BMI), body surface area (BSA)], so that these morphology characteristics could be compared between different patients.

Different from the focus on LAA volume for a specific cardiac phase, Al-Issa et al. (58) focused on the changes in LAA volume during the whole cardiac cycle. In their study, the LAA area change ratio (known as regional function) was measured by CT and used to denote the volume change, and the result showed this parameter was significantly associated with stroke risk history, where stroke/TIA group tended to have a lower area change ratio compared with the control group.

In addition to the above-mentioned characteristics, some other characteristics were also studied, such as the angle of LAA bend (59), the depth (48, 53) (see Figures 2D,E) and the number of lobes (52, 60). In these studies, the acute angle, increased depth and large number of lobes was each considered as a high-risk morphology of LAA. All these morphological characteristics suggest that the stroke risk is increased with the increasing of LAA morphology complexity. Detailed information on the associations between stroke risk and LAA morphological parameters are summarized in Table 4.

**Table 4:** Studies on the association between stroke risk and LAA morphological parameters.

**Parameters**	**References**	**No**.	**Modality**	**Criterion**	**Relation**	**Cut-off**
Orifice (area)	Adukauskaite et al. (35)	158	CT	PriS	Positive (Pos)	**-**
	Lee et al. (46)	218	CT	PriS	Pos	3.5 cm^2^
	Lee et al. (47)	66	CT	PriS	Pos	4 cm^2^
Orifice (diameter)	Khurram et al. (45)	678	CT	PriS	Negative (Neg)	**-**
	Kong et al. (41)	219	CT	PriS	Pos	**-**
	Jeong et al. (49)	88	CT	PriS	Pos	**-**
	Sakr et al. (50)	50	TEE	PriS	Pos	**-**
	Beinart et al. (48)	144	MRI	PriS/TIA	Pos	**-**
Volume	Beinart et al. (48)	144	MRI	PriS/TIA	Pos	-
	Korhonen et al. (56)	151	CT	PriS	Pos	**-**
	Jeong et al. (49)	88	CT	PriS	Pos	**-**
	Taina et al. (57)	122	CT	PriS	Pos	**-**
	Chen et al. (55)	444	TEE	DoT	Pos	8.6 ml
	Burrell et al. (54)	96	MRI	PriS	Pos	34 cm^3^
Volume change	Al-Issa et al. (58)	36	CT	PriS/TIA	Neg	**-**
Bend	Yaghi et al. (59)	408	CT	PriS/TIA	Pos	**-**
Depth	Beinart et al. (48)	144	MRI	PriS/TIA	Pos	
	Chen et al. (55)	444	TEE	DoT	Pos	**-**
	Dudzińska et al. (61)	169	CT	PriS	Pos	**-**
Lobes	Yamamoto et al. (52)	564	TEE	DoT	Pos	**-**
	Wang et al. (60)	472	TEE	DoT	Pos	**-**

PriS: Prior Stroke; TIA: Transient ischaemic attack; DoT: Detection of thrombus; TEE: Transoesophageal echocardiography; MRI: Magnetic Resonance Imaging.

To better quantify this complexity, Kaminski et al. (62) used different parameters to describe the whole LAA. In their study, different parametric schemes with various parameters were used to describe the different LAA types. In addition to Bieging et al. (63), where they assessed the LAA shape using statistical shape analysis on the “surface” of the LAA, Sanatkhani et al. (64) also performed an analysis to parametrize the LAA region's appearance based on principal components and eigen shapes. And also, Slipsager et al. (65) conducted the unsupervised clustering based on the LAA shapes, which result into two clusters: chicken wing and non-chicken wing shapes.

Although the morphology parameters are considered to be an objective approach for characterizing the LAA, the morphology parameter features are inherently isolated from the complex LAA geometry, and it lacks a comprehensive and mechanistic prospective for stroke evaluation. Under this circumstance, the hemodynamic analysis attracted the attention, for that the hemodynamic description utilizes the entire 3D dataset and is more comprehensive compared to the single or several morphological parameters. Furthermore, the study of hemodynamics is aligned with Virchow's triad used to characterize the relationship between blood flow and thrombus formation. In this regard, evaluating the stroke risk from the aspect of hemodynamics in the LAA may be an optimal approach for evaluation of stroke risk.

### LAA hemodynamics

LAA flow velocity accounts for the majority of studies on the hemodynamic characteristics. Verhorst et al. (66) firstly evaluated the stroke risk using peak LAA velocity (both emptying and filling) and concluded that peak LAA velocity may potentially identify patients at high risk for systemic embolism. In subsequent studies, both peak LAA filling and emptying velocity were found to exhibit significant difference between the stroke and non-stroke patient groups (50). In a series of related studies, the peak LAA emptying velocity (pLAAev) was then used to quantify LAA flow velocity, revealing a correlation between a lower pLAAev and a higher stroke risk (32, 39, 67–69). However, the cut-off values for pLAAev were neglected in most studies, with only a few studies calculating this value. Generally speaking, the velocity of 20 cm/s is widely accepted as the threshold for determining the stroke risk (70–72). Chen et al. (73) concluded a slightly higher cut-off value of 21.5 cm/s based on study of 307 AF patients. However, Lee et al. (47) found that 37 cm/s could be used as a prognostic threshold for stroke risk in AF patients, with a higher value of 40 cm/s in patients with low CHA_2_DS_2_-VASc scores (46). The large difference for the aforementioned values (~20 vs. ~40 cm/s) may attribute to the different placement of sample volume in the TEE measurement. In Lee et al.'s (46, 47) measurements, the sample volume was placed at the middle portion of the LAA, while the other measurements placed 1–2 cm inside of LAA.

In addition to LAA flow velocity, other hemodynamic parameters such as flow pattern and LAA ejection fraction (LAAef) have also been studied. For LAA flow pattern, Garciafernandez et al. (74) identified three different flow patterns and studied their association with stroke risk. Their results revealed that the flow pattern characterized by the absence of identifiable flow waves was associated with a higher prevalence of LAA spontaneous contrast (higher stroke risk). Regarding LAAef, consensus was reached that the LAAef is negatively related with the stroke risk (75, 76). Specifically speaking, Park et al. (76) retrospectively studied 176 paroxysmal AF patients, and the result revealed that LAAef was significantly lower in the stroke group compared with the non-stroke group. LAA emptying fraction assessed by the feature-tracking echocardiographic method was put forward by Iwama et al. (75) who found that LAA emptying fraction was significantly reduced in AF patients with thrombus compared to those without thrombus. However, the cut-off values for LAAef were not determined in the studies, which should be considered in the future research. Studies on association of stroke risk and LAA hemodynamic parameters are summarized in Table 5.

**Table 5:** Studies on association between stroke risk and LAA hemodynamic parameters.

**Parameters**	**References**	**Patients**	**Criterion**	**Modality**	**Relation**	**Cut-off**
Peak velocities	Patrick et al. (66)	54	PriS	TEE	Negative (Neg)	-
	Sakr et al. (50)	50	PriS	TEE	Neg	-
pLAAev	Goldman et al. (68)	721	Risk features	TEE	Neg	-
	Lee et al. (32)	360	PriS	TEE	Neg	-
	Lee et al. (39)	255	TIA/ PriS	TEE	Neg	-
	Taguchi et al. (69)	32	PriS	TEE	Neg	-
	Bernhardt et al. (67)	271	SEC	TEE	Neg	-
	Kamp et al. (70)	88	Prior events	TEE	Neg	20 cm/s
	Negrotto et al. (72)	306	DoT	TEE	Neg	20 cm/s
	Miyazaki et al. (71)	88	Embolic event*	TEE	Neg	20 cm/s
	Lee et al. (47)	218	PriS	TEE	Neg	37 cm/s
	Lee et al. (46)	279	PriS	TEE	Neg	40 cm/s
	Chen et al. (73)	307	DoT	TEE	Neg	21.5 cm/s
Flow pattern	Garciafernandez et al. (74)	39	SEC/DoT	TEE	TypeIII	-
LAAef	Park et al. (76)	176	PriS /TIA	TEE	Neg	-
	Iwama et al. (75)	142	DoT	TEE	Neg	-

TEE, Transoesophageal echocardiography; MRI, Magnetic Resonance Imaging; PriS, Prior Stroke; TIA, Transient ischemic attack; SEC, Spontaneous echo contrast; DoT: Detection of thrombus.

Although evaluation of stroke risk by means of LAA hemodynamic parameters is particularly convincing, the availability of direct clinical measurements of these parameters is often limited. Alternatively, computational fluid dynamics (CFD) method has been used to obtain these parameters. CFD is a numerical analysis approach for predicting the behavior of fluid flows whereby the hemodynamic details of LA and LAA can be obtained and visualized. Utilizing the CFD method, Gracia-Isla et al. (77) not only obtained the velocity profile located at the LAA orifice, but also calculated the other hemodynamic descriptors such as time-averaged wall shear stress (TAWSS), oscillatory shear index (OSI), endothelial cell activation potential (ECAP), relative residence time (RRT), and vortex structures in four different LAA models.

**Figure 4:**
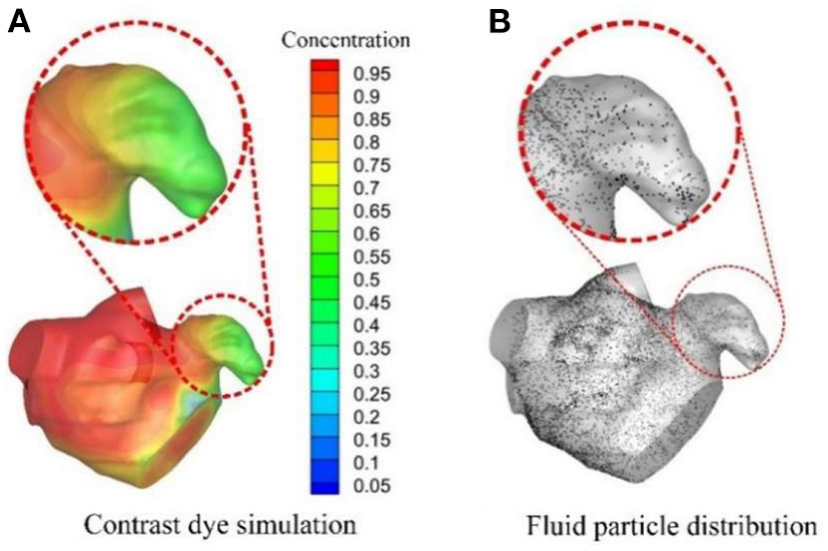
CFD analysis for evaluating thromboembolic risk in LAA. **(A)** Contrast dye simulations and **(B)** Fluid particle distributions.

Based on CFD, some other studies applied more intuitive and quantitative methods in evaluating thrombus formation. Bosi et al. (78) used contrast dye to simulate blood stasis in the LAA and then evaluated the risk of thrombus formation in the LAA (Figure 4A). Additionally, Masci et al. (79, 80) and Fang et al. (81) adopted a fluid particle distribution simulation in analyzing thrombus formation risk (Figure 4B). Similarly, Sanatkhani et al. put forward a mean residence time of blood-borne particles (82) and asymptotic concentration remaining (83) inside LAA for evaluating thrombus formation. In a further study, Yan et al. (84) applied a numerical thrombus model presented by Menichini et al. (85) to predict thrombus formation risk. This thrombus model included not only the flow characteristics, but also the biomedical characteristics of platelets and coagulation.

In addition to studies assumed the boundary walls to be rigid to simulate the idealized AF conditions (77, 78, 86–88). Different ways were applied to realize the wall motion, several studies applied the immersed boundary analysis to fulfill the fluid-structure interactions, where the walls were moved with the impact from the fluid flows (89–91). Followingly, with the lack of knowledge in the material and mechanical properties for heart walls, several other studies used a four-dimensional displacement vector field directly generated from the medical images, and the displacement filed was then applied to the LA structure to move the walls (80, 92–94). Meanwhile, with the lack in the dynamic medical images, several other studies also fulfill the wall motion with the displacements generated form literature (95, 96). To better compare the difference between the rigid walls and moving walls simulation, Duenas-Pamplona et al. (97) performed a comprehensive comparation between the two type simulations, and they found that the rigid wall model can lead to a poor approximation in some cases.

Although several hemodynamic parameters proposed by the CFD models rely on numerical simulations to evaluate thrombus formation risk. further research is required to validate their potential application in stroke risk evaluation in patients.

## Discussion

### Intra-group relationship

**Figure 5:**
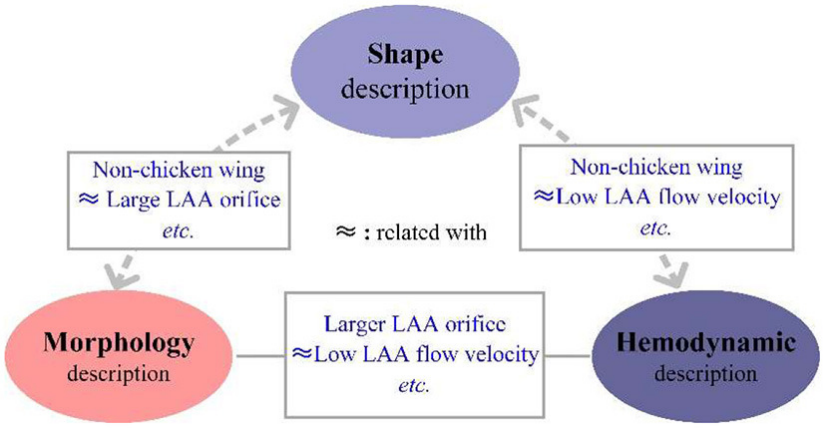
The relationship among different characteristics.

As illustrated in the former section, shape, morphology and hemodynamic parameters of the LAA are all validated to be highly associated with the risk of stroke. Several studies have pointed out the subjectivity of the shape classifications and the advantages of morphology and hemodynamic descriptors as objective classifiers, which have led to studies of relationships among these parameters. A number of these studies have established that the non-chicken wing type LAA is usually associated with a larger LAA orifice (32, 33) and higher lobe count (60). Studies on the relationships between shape and hemodynamic parameters revealed that peak LAA flow velocity exhibited significant difference among LAA shapes (72, 78) with a low LAA flow velocity associated with the non-chicken wing LAA type (98) and vice versa (33, 99). In addition, several other studies have identified the relationships between the hemodynamic and morphological parameters. In a multi-linear regression analysis, LAA morphology was found to be the significant determinant of LAA flow velocity (99). In other studies, flow velocity was shown to be negatively correlated with orifice size, and many studies found that a greater orifice size usually resulted in decreased flow velocity (32, 47, 53). CFD was also applied in studies relevant to morphology parameters in which LAA length, orifice area, tortuosity, etc. were studied for their relationship with hemodynamic parameters. Masci et al. (79) concluded that even qualitatively simple LAA morphology could lead to a high probability of thrombus formation. The summarized intra-group relationships are illustrated in the Figure 5.

The relationships among the shape, morphology and hemodynamic parameters highlight the importance of LAA in stroke evaluation, which motivates further studies to find and evaluate the key LAA features.

### Imaging modalities

As shown in the Tables 2, 4, 5, different imaging modalities were used for the evaluation of LAA shape, morphology and hemodynamic parameters, namely the TEE, cardiac CT and MRI modalities.

#### TEE

Compared with other imaging modalities, echocardiography can obtain the image at low cost and with high efficiency, so it is a widely used method in clinics. During the imaging procedure, the probe of TEE is close to the heart with high frequency, and therefore TEE can be used not only to assess the LAA morphology, but also to screen out the thrombus and spontaneous echo contrast (SEC) in the LA/LAA (100), and it is considered as the gold standard modality for detecting the LAA thrombi (100, 101). Additionally, TEE is superior in time resolution, and so it can obtain the LAA hemodynamic parameters (e.g.,: LAA flow profile) within the continuous cardiac cycles (102). Beside the above-mentioned applications, TEE is also applied in the guidance and evaluation during the LAA occlusion operation in most centers, for that TEE allows the real-time imaging, without ionizing radiation or contrast medium. Under this circumstance, TEE is considered as the conventional gold-standard imaging modality for LAA occlusion (103). However, TEE may not be applicable for some cases and it is considered to be a semi-invasive test that carries a small risk to the patient, although very rare, some complications can be life threatening (104, 105).

All in all, TEE is widely applied in the clinical practice related with LAA with its efficient, clear and low-cost imaging procedure. And this imaging modality with high time-resolution is applicable in the shape, morphology and especially the hemodynamic evaluations for the LAA.

#### Cardiac CT

Compared with TEE, cardiac CT offers superior spatial resolution. Its high-quality multi-planar and 3D reconstruction would do much help in the characterization of the LAA anatomy and accurate sizing (31). Therefore, CT has been considered as the gold standard for visualizing the LAA (106, 107), and it is widely applied in the evaluation of shape and morphology parameters. Meanwhile, some studies have also considered cardiac CT with a delayed imaging as a useful modality for the detection of LAA thrombi (108), and therefore it is routinely performed to screen the LA/LAA thrombi before the AF ablation procedure in clinics. However, compared to TEE, CT imaging modality is limited in the time resolution, it cannot get the real-time visualization of the hemodynamics and LAA structure with the cardiac cycle. To strike the deficiency in the time resolution, it is worth mentioning a new CT-based method proposed by Morelos-Guzman (109), which can calculate the flow velocity with the scans. It is a novel method that can expend the CT application to the hemodynamic parameter evaluation.

In general, cardiac CT is limited in the time resolution, but its high spatial resolution contributes to a wide application in the shape and morphology evaluation for LAA.

#### Cardiac MRI

Cardiac MRI is a noninvasive imaging modality for the structure and hemodynamic evaluation. It has excellent tissue characterization capacity but a lower spatial resolution when compared to the cardiac CT (110). Meanwhile, it is often used to delineate the anatomy of the left atrium but conferring no ionizing radiation to patients. Additionally, MRI can measure the LAA flow velocity and shows a good correlation with TEE (111), and MRI shows the good sensitivity and specificity in evaluating the LAA thrombi (110), thus, it has the potential to be a useful imaging modality for the detection of LAA thrombi. However, similar with CT, the real-time visualization of LAA is not permitted and it is not applicable for patients with pacemakers.

Generally speaking, though the MRI modality is noninvasive and can obtain the shape, morphology and hemodynamic parameters of the LAA, the present number of its application scenarios in LAA are small, most for the patients unsuitable for TEE and CT (110). Its potential capabilities in LAA related evaluation will be strongly stimulated with the high-resolution MRI in the future applications.

### Perspectives

Stroke prevention is the cornerstone of AF management and the anticoagulation decision is currently based on the CHA_2_DS_2_-VASc score scheme. This scheme first became part of guideline medical therapy as part of the 2012 European Society of Cardiology updated guidelines (112) as well as the 2014 American Heart Association/American College of Cardiology/Heart Rhythm Society (AHA/ACC/HRS) guideline for the management of patients with AF (113). Both the European and US guidelines specifically recommend risk stratification with the CHA_2_DS_2_-VASc score, and delineate that anticoagulation may be considered for males with a score of 1 and females with a score of 2 (113, 114). However, CHA_2_DS_2_-VASc score does not take into account paroxysmal vs. non-paroxysmal AF as well as duration of paroxysmal AF episodes. Even patients with low CHA_2_DS_2_-VASc score may suffer a stroke. The score also fails to take into consideration the structure (31, 115) and the hemodynamic characteristics for the LAA (37, 116, 117) in addition to other clinical markers (118–120). Thus, additional LAA features and clinical markers may further risk stratify stroke prevention for patients with AF. These two additional predictors may improve the sensitivity and specificity of LAA-related stroke risk.

Meanwhile, with the requirement of large data bank processing, several potential tools (or algorithms) could be utilized for the LAA related studies:

#### Computer aided pre-processing

The pre-processing of image segmentation and reconstruction are the foundation for shape and morphology evaluation, while the manual segmentation and reconstruction for a patient-specific data is time-consuming. In this regard, some auto or semi-auto segmentation and reconstruction algorithms were put forward (121–124) to improve the efficiency. Besides the study for the efficient segmentation and reconstruction algorithms, there is also a need for the image-based measurement algorithms. With the measurement algorithms, not only the time of the measurement can be greatly reduced, but also the reproducibility will be improved. In this regard, studies that focus on the auto or semi-auto measurement, like Leventic et al.'s (125) work, should be encouraged in future studies. Clinical studies are also needed to validate the developed algorithms.

#### CFD analysis

Blood stasis is one of the three conditions in Virchow's triad, the study of blood stasis requires a comprehensive characterization of the local hemodynamics, and the clinical measurement of echocardiography is currently the main tool to explore the blood flow patterns and velocity profiles. While the echocardiography cannot fully characterize the complexity of 4D blood flow patterns, new imaging techniques like 4D flow MRI or blood speckle tracking are promising but their capability to capture the low flow velocities related to thrombus formation is still unclear. As an alternative, CFD poses a great potential to thoroughly assess the hemodynamics inside the LA/LAA, and several studies have been performed to analyze the hemodynamic parameters in the LAA in relation to thrombogenic risk (72, 80–82, 84).

However, these CFD analysis uses various settings for boundary conditions, LA wall behavior, mesh resolution and etc., and different modeling strategies were used. It has to admit that the optimal strategy is difficult to identify due to the absence of joint benchmark studies with reliable ground truth (126). Meanwhile, there is no consensus on the most appropriate metrics for the quantification of the CFD result. Therefore, the consensus for the appropriate metrics needed to be reached in the future studies. Based on the consensus, the modeling pipelines would be proposed for improving the capacity to process the increased number specific cases. Moreover, these CFD analysis should be validated with reliable ground truth to form the benchmark studies for optimal strategy in future work, and Duenas-Pamplona et al. (127) proposed an *in-vitro* flow equipment to validate there CFD models. Finally, it is worth to mention the work performed by Morales et al. (96), where they performed a deep learning surrogate models of CFD analysis for thrombus formation risk evaluation in the LAA.

## Conclusion

Stroke prevention remains a major goal in patients in AF. Significant evidence indicates that the assessment of LAA structural and hemodynamic parameters have meaningful implications for the stroke risk evaluation. Future work is needed to understand the role of LAA structural (shape and morphology) and hemodynamic parameters on stratification of stroke risk, especially in patients with low CHA_2_DS_2_-VASc scores. Computer-aided tools will become instrumental in the stroke evaluation with these LAA-related parameters.

## Author contributions

ZL and LZ designed and supervised the study. RF and YL reviewed the literature and wrote the original manuscript. JW and ZW helped with the literature search. JA and CC modified the article. All authors contributed to the article and approved the submitted version.

## Funding

This work is partially supported by the National Natural Science Foundation of China (11972118, 61821002, 12172089), Postgraduate Research & Practice Innovation Program of Jiangsu Province (No.SJCX20_0012) and the Fundamental Research Funds for the Central Universities (3207032103D).

## Conflict of interest

The authors declare that the research was conducted in the absence of any commercial or financial relationships that could be construed as a potential conflict of interest.

## Publisher's note

All claims expressed in this article are solely those of the authors and do not necessarily represent those of their affiliated organizations, or those of the publisher, the editors and the reviewers. Any product that may be evaluated in this article, or claim that may be made by its manufacturer, is not guaranteed or endorsed by the publisher.

## References

[1] Go AS Hylek EM Phillips KA Chang YC Henault LE Selby JV. Prevalence of diagnosed atrial fibrillation in adults - National implications for rhythm management and stroke prevention: the Anticoagulation and Risk Factors in Atrial Fibrillation (ATRIA) study. JAMA. (2001) 285:2370–5. 10.1001/jama.285.18.2370 11343485

[2] Andrade J Khairy P Dobrev D Nattel S. The clinical profile and pathophysiology of atrial fibrillation relationships among clinical features, epidemiology, and mechanisms. Circ Res. (2014) 114:1453–68. 10.1161/CIRCRESAHA.114.303211 24763464

[3] Camm AJ Kirchhof P Lip GYH Schotten U Savelieva IS. Guidelines for the management of atrial fibrillation The Task Force for the Management of Atrial Fibrillation of the European Society of Cardiology (ESC). Eur Heart J. (2010) 31:2369–429. 10.1093/eurheartj/ehq278 20802247

[4] McManus DD Rienstra M Benjamin EJ. An update on the prognosis of patients with atrial fibrillation. Circulation. (2012) 126:E143–6. 10.1161/CIRCULATIONAHA.112.129759 22949543 PMC3678907

[5] January CT Wann LS Alpert JS Calkins H Cigarroa JE Cleveland JC. 2014 AHA/ACC/HRS guideline for the management of patients with atrial fibrillation: executive summary. J Am Coll Cardiol. (2014) 64:2246–80. 10.1016/j.jacc.2014.03.021 24685669

[6] Virani SS Alonso A Benjamin EJ Bittencourt MS Callaway CW Carson AP. Heart Disease and Stroke Statistics-2020 Update: A Report From the American Heart Association. Circulation. (2020) 141:E139–E596. 10.1161/CIR.0000000000000746 31992061

[7] Pistoia F Sacco S Tiseo C Degan D Ornello R Carolei A. The epidemiology of atrial fibrillation and stroke. Cardiol Clin. (2016) 34:255–63. 10.1016/j.ccl.2015.12.002 27150174

[8] Wilke T Groth A Mueller S Pfannkuche M Verheyen F Linder R. Incidence and prevalence of atrial fibrillation: an analysis based on 8.3 million patients. Europace. (2013) 15:486–93. 10.1093/europace/eus333 23220354

[9] Morin DP Bernard ML Madias C Rogers PA Thihalolipavan S Estes NAM. The State of the Art: Atrial Fibrillation Epidemiology, Prevention, and Treatment. Mayo Clin Proc. (2016) 91:1778–810. 10.1016/j.mayocp.2016.08.022 27825618

[10] Staerk L Sherer JA Ko D Benjamin EJ Helm RH. Atrial fibrillation epidemiology, pathophysiology, and clinical outcomes. CircRes. (2017) 120:1501–17. 10.1161/CIRCRESAHA.117.309732 28450367 PMC5500874

[11] Bjorck S Palaszewski B Friberg L Bergfeldt L. Atrial fibrillation, stroke risk, and warfarin therapy revisited a population-based study. Stroke. (2013) 44:3103–8. 10.1161/STROKEAHA.113.002329 23982711

[12] Naccarelli GV Varker H Lin J Schulman KL. Increasing prevalence of atrial fibrillation and flutter in the United States. Am J Cardiol. (2009) 104:1534–9. 10.1016/j.amjcard.2009.07.022 19932788

[13] Hart RG Pearce LA Halperin JL Hylek EM Albers GW Anderson DC. Comparison of 12 risk stratification schemes to predict stroke in patients with nonvalvular atrial fibrillation. Stroke. (2008) 39:1901–10. 10.1161/STROKEAHA.107.501825 18420954

[14] Ganjehei L Massumi A Razavi M Rasekh A. Stroke prevention in nonvalvular atrial fibrillation. Texas Heart Inst J. (2011) 38:350–2. PMC3147190 21841857

[15] Petersen P. Thromboembolic complications in atrial-fibrillation. Stroke. (1990) 21:4–13. 10.1161/01.STR.21.1.4 2405547

[16] Wolf PA Abbott RD Kannel WB. Atrial-fibrillation as an independent risk factor for stroke - the framingham-study. Stroke. (1991) 22:983–8. 10.1161/01.STR.22.8.983 1866765

[17] Lip GYH. Stroke and bleeding risk assessment in atrial fibrillation: when, how, and why? Eur Heart J. (2013) 34:1041–9. 10.1093/eurheartj/ehs435 23257951

[18] Okuyama H Hirono O Liu L Takeishi Y Kayama T Kubota I. Higher levels of serum fibrin-monomer reflect hypercoagulable state and thrombus formation in the left atrial appendage in patients with acute ischemic stroke. Circ J. (2006) 70:971–6. 10.1253/circj.70.971 16864927

[19] Yaghi S Song C Gray WA Furie KL Elkind MS Kamel H. Left atrial appendage function and stroke risk. Stroke. (2015) 46:3554–9. 10.1161/STROKEAHA.115.011273 26508750 PMC4659748

[20] Beigel R Wunderlich NC Ho SY Arsanjani R Siegel RJ. The left atrial appendage: anatomy, function, and noninvasive evaluation. JACC Cardiovasc Imaging. (2014) 7:1251–65. 10.1016/j.jcmg.2014.08.009 25496544

[21] Sharma S Devine W Anderson RH Zuberbuhler JR. The determination of atrial arrangement by examination of appendage morphology in 1842 heart specimens. Br Heart J. (1988) 60:227–31. 10.1136/hrt.60.3.227 3179139 PMC1216558

[22] Ucerler H Ikiz ZAA Ozgur T. Human left atrial appendage anatomy and overview of its clinical significance. Anat J Cardiol. (2013) 13:566–72. 10.5152/akd.2013.181 23886901

[23] Reddy VY Sievert H Halperin J Doshi SK Buchbinder M Neuzil P. Percutaneous left atrial appendage closure vs warfarin for atrial fibrillation a randomized clinical trial. JAMA. (2014) 312:1988–98. 10.1001/jama.2014.15192 25399274

[24] Tuccillo B Stumper O Hess J Vansuijlen RJ Bos E Roelandt J. Transesophageal echocardiographic evaluation of atrial morphology in children with congenital heart-disease. Eur Heart J. (1992) 13:223–31. 10.1093/oxfordjournals.eurheartj.a060151 1555621

[25] Boyle PM del Alamo JC Akoum N. Fibrosis, atrial fibrillation and stroke: clinical updates and emerging mechanistic models. Heart. (2021) 107:99–105. 10.1136/heartjnl-2020-317455 33097562

[26] Wang Y Biase LDi Horton RP Nguyen T Morhanty P Natale A. Left atrial appendage studied by computed tomography to help planning for appendage closure device placement. J Cardiovasc Electrophysiol. (2010) 21:973–82. 10.1111/j.1540-8167.2010.01814.x 20550614

[27] Koplay M Erol C Paksoy Y Kivrak AS Ozbek S. An investigation of the anatomical variations of left atrial appendage by multidetector computed tomographic coronary angiography. Eur J Radiol. (2012) 81:1575–80. 10.1016/j.ejrad.2011.04.060 21592706

[28] Shi AW Chen ML Yang B Cao KJ Kong XQ. A morphological study of the left atrial appendage in chinese patients with atrial fibrillation. J Int Med Res. (2012) 40:1560–7. 10.1177/147323001204000436 22971509

[29] Beutler DS Gerkin RD Loli AI. The morphology of left atrial appendage lobes: a novel characteristic naming scheme derived through three-dimensional cardiac computed tomography. World J Cardiovasc Surg. (2014) 4:17–24. 10.4236/wjcs.2014.43004

[30] Jun W Yi X Xiaomei Z Xin T Weiwei H Haibin S. The value of left atrial parameters measured on cardiac CT in predicting stroke in patients with atrial fibrillation (in chinese). Nat Sci. (2019) 39:136–40. 10.7655/NYDXBNS20190128

[31] Biase LDi Santangeli P Anselmino M Mohanty P Salvetti I Gili S. Does the left atrial appendage morphology correlate with the risk of stroke in patients with atrial fibrillation? Results from a multicenter study. J Am Coll Cardiol. (2012) 60:531–8. 10.1016/j.jacc.2012.04.032 22858289

[32] Lee JM Seo J Uhm JS Kim YJ Lee HJ Kim JY. Why is left atrial appendage morphology related to strokes? An analysis of the flow velocity and orifice size of the left atrial appendage. J Cardiovasc Electrophysiol. (2015) 26:922–7. 10.1111/jce.12710 25959871

[33] Lupercio F Ruiz JC Briceno DF Romero J Villablanca PA Berardi C. Left atrial appendage morphology assessment for risk stratification of embolic stroke in patients with atrial fibrillation: a meta-analysis. Heart Rhythm. (2016) 13:1402–9. 10.1016/j.hrthm.2016.03.042 27016474

[34] Basu-Ray I Sudhakar D Schwing G Monlezun D Zhang L Shah SK. Complex left atrial appendage morphology is an independent risk factor for cryptogenic ischemic stroke. Front Cardiovasc Med. (2018) 5:8. 10.3389/fcvm.2018.00131 30460239 PMC6232927

[35] Adukauskaite A Barbieri F Senoner T Plank F Knoflach M Boehme C. Left atrial appendage morphology and left atrial wall thickness are associated with cardio-embolic stroke. J Clin Med. (2020) 9:9. 10.3390/jcm9123944 33291376 PMC7762068

[36] Anselmino M Scaglione M Biase LD Gili S Santangeli P Corsinovi L. Left atrial appendage morphology and silent cerebral ischemia in patients with atrial fibrillation. Heart Rhythm. (2014) 11:2–7. 10.1016/j.hrthm.2013.10.020 24120872

[37] Deng BQ Nie RQ Qiu Q Wei YL Liu YM Lv HL. 3D transesophageal echocardiography assists in evaluating the morphology, function, and presence of thrombi of left atrial appendage in patients with atrial fibrillation. Ann Transl Med. (2021) 9:13. 10.21037/atm-21-1981 34164510 PMC8184463

[38] Kimura T Takatsuki S Inagawa K Katsumata Y Nishiyama T Nishiyama N. Anatomical characteristics of the left atrial appendage in cardiogenic stroke with low CHADS2 scores. Heart Rhythm. (2013) 10:921–5. 10.1016/j.hrthm.2013.01.036 23384894

[39] Lee Y Park HC Lee Y Kim SG. Comparison of morphologic features and flow velocity of the left atrial appendage among patients with atrial fibrillation alone, transient ischemic attack, and cardioembolic stroke. Am J Cardiol. (2017) 119:1596–604. 10.1016/j.amjcard.2017.02.016 28364953

[40] Smit JM Simon J El Mahdiui M Szaraz L van Rosendael PJ Kolassvary M. Anatomical characteristics of the left atrium and left atrial appendage in relation to the risk of stroke in patients with vs. without atrial fibrillation. Circ Arrhythm Electrophysiol. (2021) 14:10. 10.1161/CIRCEP.121.009777 34279121

[41] Kong B Liu Y Hu H Wang L Fan Y Mei Y. Left atrial appendage morphology in patients with atrial fibrillation in China: implications for stroke risk assessment from a single center study. Chin Med J. (2014) 127:4210–4. 10.3760/cma.j.issn.0366-6999.20141520 25533823

[42] Du H Bi K Xu LS Chen F Xiong WF Wang Y. Analysis of risk factors for thrombosis of the left atrium/left atrial appendage in patients with non-valvular atrial fibrillation. Cardiovasc J Afr. (2021) 32:116–22. 10.5830/CVJA-2019-071 33950066 PMC8756031

[43] Yaghi S Chang AD Hung P Mac Grory B Collins S Gupta A. Left atrial appendage morphology and embolic stroke of undetermined source: a cross-sectional multicenter pilot study. J Stroke Cerebrovasc Dis. (2018) 27:1497–501. 10.1016/j.jstrokecerebrovasdis.2017.12.036 29398537

[44] Wu LM Liang EP Fan SY Zheng LH Du ZP Liu SY. Relation of left atrial appendage morphology determined by computed tomography to prior stroke or to increased risk of stroke in patients with atrial fibrillation. Am J Cardiol. (2019) 123:1283–6. 10.1016/j.amjcard.2019.01.024 30709597

[45] Khurram IM Dewire J Mager M Maqbool F Zimmerman SL Zipunnikov V. Relationship between left atrial appendage morphology and stroke in patients with atrial fibrillation. Heart Rhythm. (2013) 10:1843–9. 10.1016/j.hrthm.2013.09.065 24076444

[46] Lee JM Kim JB Uhm JS Pak HN Lee MH Joung B. Additional value of left atrial appendage geometry and hemodynamics when considering anticoagulation strategy in patients with atrial fibrillation with low CHA(2)DS(2)-VASc scores. Heart Rhythm. (2017) 14:1297–301. 10.1016/j.hrthm.2017.05.034 28559088

[47] Lee JM Shim J Uhm JS Kim YJ Lee HJ Pak HN. Impact of increased orifice size and decreased flow velocity of left atrial appendage on stroke in nonvalvular atrial fibrillation. Am J Cardiol. (2014) 113:963–9. 10.1016/j.amjcard.2013.11.058 24462064

[48] Beinart R Heist EK Newell JB Holmvang G Ruskin JN Mansour M. Left atrial appendage dimensions predict the risk of stroke/TIA in patients with atrial fibrillation. J Cardiovasc Electrophysiol. (2011) 22:10–5. 10.1111/j.1540-8167.2010.01854.x 20662984

[49] Jeong WK Choi JH Son JP Lee S Lee MJ Choe YH. Volume and morphology of left atrial appendage as determinants of stroke subtype in patients with atrial fibrillation. Heart Rhythm. (2016) 13:820–7. 10.1016/j.hrthm.2015.12.026 26707792

[50] Sakr SA El-Rasheedy WA Ramadan MM El-Menshawy I Mahfouz E Bayoumi M. Association between left atrial appendage morphology evaluated by trans-esophageal echocardiography and ischemic cerebral stroke in patients with atrial fibrillation. Int Heart J. (2015) 56:329–34. 10.1536/ihj.14-374 25912903

[51] Korsholm K Berti S Iriart X Saw J Wang DD Cochet H. Expert recommendations on cardiac computed tomography for planning transcatheter left atrial appendage occlusion. JACC. (2020) 13:277–92. 10.1016/j.jcin.2019.08.054 31678086

[52] Yamamoto M Seo Y Kawamatsu N Sato K Sugano A Machino-Ohtsuka T. Complex left atrial appendage morphology and left atrial appendage thrombus formation in patients with atrial fibrillatio*n*. Circ Cardiovasc Imaging. (2014) 7:337–43. 10.1161/CIRCIMAGING.113.001317 24523417

[53] Chen L Xu CJ Chen WS Zhang CQ. Left atrial appendage orifice area and morphology is closely associated with flow velocity in patients with nonvalvular atrial fibrillation. BMC Cardiovasc Disord. (2021) 21:13. 10.1186/s12872-021-02242-9 34530731 PMC8443967

[54] Burrell LD Horne BD Anderson JL Muhlestein JB Whisenant BK. Usefulness of left atrial appendage volume as a predictor of embolic stroke in patients with atrial fibrillation. Am J Cardiol. (2013) 112:1148–52. 10.1016/j.amjcard.2013.05.062 23827402

[55] Chen ZX Bai WJ Li C Wang H Tang H Qin YP. Left atrial appendage parameters assessed by real-time three dimensional transesophageal echocardiography predict thromboembolic risk in patients with nonvalvular atrial fibrillation. J Ultrasound Med. (2017) 36:1119–28. 10.7863/ultra.16.05070 28233335

[56] Korhonen M Muuronen A Arponen O Mustonen P Hedman M Jakala P. Left atrial appendage morphology in patients with suspected cardiogenic stroke without known atrial fibrillation. PLoS ONE. (2015) 10:12. 10.1371/journal.pone.0118822 25751618 PMC4353716

[57] Taina M Vanninen R Hedman M Jakala P Karkkainen S Tapiola T. Left atrial appendage volume increased in more than half of patients with cryptogenic stroke. PLoS ONE. (2013) 8:8. 10.1371/journal.pone.0079519 24223960 PMC3817123

[58] Al-Issa A Inoue Y Cammin J Tang Q Nazarian S Calkins H. Regional function analysis of left atrial appendage using motion estimation CT and risk of stroke in patients with atrial fibrillation. Eur Heart J Cardiovasc Imaging. (2016) 17:788–96. 10.1093/ehjci/jev207 26341293 PMC4907380

[59] Yaghi S Chang AR Ignacio G Scher E Panda N Chu AT. Left atrial appendage morphology improves prediction of stagnant flow and stroke risk in atrial fibrillation. Circ Arrhythm Electrophysiol. (2020) 13:3. 10.1161/CIRCEP.119.008074 31986073 PMC7446933

[60] Wang F Zhu MY Wang XY Zhang W Su Y Lu YY. Predictive value of left atrial appendage lobes on left atrial thrombus or spontaneous echo contrast in patients with non-valvular atrial fibrillation. BMC Cardiovasc Disord. (2018) 18:11. 10.1186/s12872-018-0889-y 30064363 PMC6069846

[61] Dudzińska-Szczerba K Michałowska I Piotrowski R Sikorska A Paszkowska A Stachnio U. Assessment of the left atrial appendage morphology in patients after ischemic stroke — The ASSAM study. Int J Cardiol. (2021) 330:65–72. 10.1016/j.ijcard.2021.01.001 33524464

[62] Kaminski R Kosinski A Brala M Piwko G Lewicka E Dabrowska-Kugacka A. Variability of the left atrial appendage in human hearts. PLoS ONE. (2015) 10:9. 10.1371/journal.pone.0141901 26544191 PMC4636143

[63] Bieging ET Morris A Chang L Dagher L Marrouche NF Cates J. Statistical shape analysis of the left atrial appendage predicts stroke in atrial fibrillation. Int J Cardiovasc Imaging. (2021) 37:2521–7. 10.1007/s10554-021-02262-8 33956285 PMC8316337

[64] Sanatkhani S Menon PG. Generative statistical modeling of left atrial appendage appearance to substantiate clinical paradigms for stroke risk stratification. In: Conference on Medical Imaging - Image Processing. Houston, TX. (2018).

[65] Slipsager JM Juhl KA Sigvardsen PE Kofoed KF De Backer O Olivares AL. Statistical shape clustering of left atrial appendages. In: Pop M Sermesant M Zhao J Li S McLeod K Young A., editors. Statistical Atlases and Computational Models of the Heart. Atrial Segmentation and LV Quantification Challenges. Shenzhen: Springer International Publishing. (2019), p. 32–39.

[66] Verhorst PMJ Kamp O Visser CA Verheugt FWA. Left Atrial Appendage Flow Velocity Assessment Using Transesophageal Echocardiography In Nonrheumatic Atrial-Fibrillation And Systemic Embolism. Am J Cardiol. (1993) 71:192–6. 10.1016/0002-9149(93)90737-W 8421982

[67] Bernhardt P Schmidt H Hammerstingl C Luderitz B Omran H. Patients with atrial fibrillation and dense spontaneous echo contrast at high risk - a prospective and serial follow-up over 12 months with transesophageal echocardiography and cerebral magnetic resonance imaging. J Am Coll Cardiol. (2005) 45:1807–12. 10.1016/j.jacc.2004.11.071 15936610

[68] Goldman ME Pearce LA Hart RG Zabalgoitia M Asinger RW Safford R.. Stroke Prevention Atrial, Pathophysiologic correlates of thromboembolism in nonvalvular atrial fibrillation: I Reduced flow velocity in the left atrial appendage (The Stroke Prevention in Atrial Fibrillation SPAF-III study). J Am Soc Echocardiogr. (1999) 12:1080–7. 10.1016/S0894-7317(99)70105-7 10588784

[69] Taguchi Y Takashima S Hirai T Fukuda N Ohara K Nakagawa K. Significant impairment of left atrial function in patients with cardioembolic stroke caused by paroxysmal atrial fibrillation. Intern Med. (2010) 49:1727–32. 10.2169/internalmedicine.49.3580 20720349

[70] Kamp O Verhorst PMJ Welling RC Visser CA. Importance of left atrial appendage flow as a predictor of thromboembolic events in patients with atrial fibrillation. Eur Heart J. (1999) 20:979–85. 10.1053/euhj.1998.1453 10361051

[71] Miyazaki S Ito T Suwa M Nakamura T Kobashi A Kitaura Y. Role of transesophageal echocardiography in the prediction of thromboembolism in patients with chronic nonvalvular atrial fibrillation. Jpn Circ J Engl Ed. (2001) 65:874–8. 10.1253/jcj.65.874 11665791

[72] Negrotto SM Lugo RM Metawee M Kanagasundram AN Chidsey G Baker MT. Left atrial appendage morphology predicts the formation of left atrial appendage thrombus. J Cardiovasc Electrophysiol. (2021) 32:1044–52. 10.1111/jce.14922 33512055

[73] Chen L Zinda A Rossi N Han XJ Sprankle S Bullock-Palmer R. A new risk model of assessing left atrial appendage thrombus in patients with atrial fibrillation - Using multiple clinical and transesophageal echocardiography parameters. Int J Cardiol. (2020) 314:60–3. 10.1016/j.ijcard.2020.04.039 32305560

[74] Garciafernandez MA Torrecilla EC Roman DS Azevedo J Bueno H Moreno MM. Left atrial appendage doppler flow patterns - implications on thrombus formation. Am Heart J. (1992) 124:955–61. 10.1016/0002-8703(92)90978-5 1529906

[75] Iwama M Kawasaki M Tanaka R Ono K Watanabe T Hirose T. Left atrial appendage emptying fraction assessed by a feature-tracking echocardiographic method is a determinant of thrombus in patients with nonvalvular atrial fibrillation. J Cardiol. (2012) 59:329–36. 10.1016/j.jjcc.2012.01.002 22342529

[76] Park HC Shin J Ban JE Choi JI Park SW Kim YH. Left atrial appendage: morphology and function in patients with paroxysmal and persistent atrial fibrillation. Int J Cardiovasc Imaging. (2013) 29:935–44. 10.1007/s10554-012-0161-y 23197275

[77] Garcia-Isla G Olivares AL Silva E Nunez-Garcia M Butakoff C Sanchez-Quintana D. Sensitivity analysis of geometrical parameters to study haemodynamics and thrombus formation in the left atrial appendage. Int J Numer Methods Biomed Eng. (2018) 34:14. 10.1002/cnm.3100 29737037

[78] Bosi GM Cook A Rai R Menezes LJ Schievano S Torii R. Computational fluid dynamic analysis of the left atrial appendage to predict thrombosis risk. Front Cardiovasc Med. (2018) 5:8. 10.3389/fcvm.2018.00034 29670888 PMC5893811

[79] Masci A Barone L Dedè L Fedele M Tomasi C Quarteroni A. The impact of left atrium appendage morphology on stroke risk assessment in atrial fibrillation: a computational fluid dynamics study. Front Physiol. (2019) 9:11. 10.3389/fphys.2018.01938 30723422 PMC6349592

[80] Masci A Alessandrini M Forti D Menghini F Dedé L Tomasi C. A proof of concept for computational fluid dynamic analysis of the left atrium in atrial fibrillation on a patient-specific basis. J Biomech Eng. (2020) 142:11. 10.1115/1.4044583 31513697

[81] Fang RX Li Y Zhang YJ Chen Q Liu QJ Li ZY. Impact of left atrial appendage location on risk of thrombus formation in patients with atrial fibrillation. Biomech Model Mechanobiol. (2021) 20:1431–43. 10.1007/s10237-021-01454-4 33755847

[82] Sanatkhani S Nedios S Menon PG Bollmann A Hindricks G Shroff SG. Subject-specific calculation of left atrial appendage blood-borne particle residence time distribution in atrial fibrillation. Front Physiol. (2021) 12:12. 10.3389/fphys.2021.633135 34045972 PMC8148016

[83] Sanatkhani S Menon PG. Relating atrial appendage flow stasis risk from computational fluid dynamics to imaging based appearance paradigms for cardioembolic risk. In: International Workshop on Bio-Imaging and Visualization for Patient-Customized Simulations (BIVPCS) / International Workshop on Point-of-Care Ultrasound - Algorithms, Hardware, and Applications (POCUS). Quebec, Canada. (2017), pp. 86–93.

[84] Yan W Qiao YH Mao YK Jiang CY Fan JR Luo K. Numerical prediction of thrombosis risk in left atrium under atrial fibrillation. Math Biosci Eng. (2020) 17:2348–60. 10.3934/mbe.2020125 32233539

[85] Menichini C Xu XY. Mathematical modeling of thrombus formation in idealized models of aortic dissection: initial findings and potential applications. J Math Biol. (2016) 73:1205–26. 10.1007/s00285-016-0986-4 27007280 PMC5055578

[86] Aguado AM Olivares AL Yague C Silva E Nunez-Garcia M Femandez-Quilez A. In silico optimization of left atrial appendage occluder implantation using interactive and modeling tools. Front Physiol. (2019) 10:237. 10.3389/fphys.2019.00237 30967786 PMC6440369

[87] Wang LF Wang ZD Fang RX Li ZY. Evaluation of stroke risk in patients with atrial fibrillation using morphological and hemodynamic characteristics. Front Cardiovasc Med. (2022) 9:8. 10.3389/fcvm.2022.842364 35571199 PMC9098797

[88] D'Alessandro N Masci A Andalo A Dede L Tomasi C Quarteroni A. Simulation of the Hemodynamic Effects of the Left Atrial Appendage Occlusion in Atrial Fibrillation: Preliminary Results, Computing in Cardiology Conference (CinC). Rimini, Italy. (2020).

[89] Zhang LT Gay M. Characterizing left atrial appendage functions in sinus rhythm and atrial fibrillation using computational models. J Biomech. (2008) 41:2515–23. 10.1016/j.jbiomech.2008.05.012 18579148

[90] Feng LY Gao H Griffith B Niederer S Luo XY. Analysis of a coupled fluid-structure interaction model of the left atrium and mitral valve. Int J Numer Methods Biomed Eng. (2019) 35:e3254. 10.1002/cnm.3254 31454470 PMC7003446

[91] Gonzalo M Garcia-Villalba Rossini L Duran E Vigneault D Martinez-Legazpi P. Non-newtonian blood rheology impacts left atrial stasis in patient-specific simulations. Int J Numer Methods Biomed Eng. (2022) 38:e3597. 10.1002/cnm.3597 35344280 PMC9189054

[92] Otani T Al-Issa A Pourmorteza A McVeigh ER Wada S Ashikaga H. A computational framework for personalized blood flow analysis in the human left atrium. Ann Biomed Eng. (2016) 44:3284–94. 10.1007/s10439-016-1590-x 26968855

[93] Qureshi A Darwish O Dillon-Murphy D Chubb H Williams S Nechipurenko D. Modelling left atrial flow and blood coagulation for risk of thrombus formation in atrial fibrillation. In: Computing in Cardiology Conference (CinC). Rimini, Italy. (2020).

[94] Garcia-Villalba M Rossini L Gonzalo A Vigneault D Martinez-Legazpi P Duran E. Demonstration of patient-specific simulations to assess left atrial appendage thrombogenesis risk. Front Physiol. (2021) 12:596596. 10.3389/fphys.2021.596596 33716763 PMC7953154

[95] Vella D Monteleone A Musotto G Bosi GM Burriesci G. Effect of the alterations in contractility and morphology produced by atrial fibrillation on the thrombosis potential of the left atrial appendage. Front Bioeng Biotechnol. (2021) 9:586041. 10.3389/fbioe.2021.586041 33718333 PMC7952649

[96] Morales X Mill J Juhl KA Olivares A Jimenez-Perez G Paulsen RR. Deep learning surrogate of computational fluid dynamics for thrombus formation risk in the left atrial appendage. In: 10th International Workshop on Statistical Atlases and Computational Modelling of the Heart (STACOM). Shenzhen, Peoples R China. (2019), pp. 157–66.

[97] Duenas-Pamplona J Garcia Garcia J Sierra-Pallares J Ferrera C Agujetas R Lopez-Minguez JR. A comprehensive comparison of various patient-specific CFD models of the left atrium for atrial fibrillation patients. Comput Biol Med. (2021) 133:104423. 10.1016/j.compbiomed.2021.104423 33957460

[98] Kishima H Mine T Ashida K Sugahara M Kodani T Masuyama T. Does left atrial appendage morphology influence left atrial appendage flow velocity? Circ J. (2015) 79:1706–11. 10.1253/circj.CJ-14-1380 25959433

[99] Fukushima K Fukushima N Kato K Ejima K Sato H Fukushima K. Correlation between left atrial appendage morphology and flow velocity in patients with paroxysmal atrial fibrillation. Eur Heart J Cardiovasc Imaging. (2016) 17:59–66. 10.1093/ehjci/jev117 25944049

[100] Manning WJ Weintraub RM Waksmonski CA Haering JM Rooney PS Maslow AD. Accuracy of transesophageal echocardiography for identifying left atrial thrombi - a prospective, intraoperative study. Ann Intern Med. (1995) 123:817-22. 10.7326/0003-4819-123-11-199512010-00001 7486462

[101] Bhagirath P van der Graaf AWM Karim R van Driel V Ramanna H. Multimodality imaging for patient evaluation and guidance of catheter ablation for atrial fibrillation - current status and future perspective. Int J Cardiol. (2014) 175:400–8. 10.1016/j.ijcard.2014.06.047 25012494

[102] Romero J Cao JJ Garcia MJ Taub CC. Cardiac imaging for assessment of left atrial appendage stasis and thrombosis. Nat Rev Cardiol. (2014) 11:470–80. 10.1038/nrcardio.2014.77 24913058

[103] Farkowski MM Jubele K Marin F Gandjbakhch E Ptaszynski P Merino JL. Diagnosis and management of left atrial appendage thrombus in patients with atrial fibrillation undergoing cardioversion or percutaneous left atrial procedures: results of the European Heart Rhythm Association survey. Europace. (2020) 22:162–9. 10.1093/europace/euz257 31501852

[104] Romero J Husain SA Kelesidis I Sanz J Medina HM Garcia MJ. Detection of left atrial appendage thrombus by cardiac computed tomography in patients with atrial fibrillation a meta-analysis. Circ Cardiovasc Imaging. (2013) 6:185–94. 10.1161/CIRCIMAGING.112.000153 23406625

[105] Nakajima H Seo Y Ishizu T Yamamoto M Machin T Harimura Y. Analysis of the left atrial appendage by three-dimensional transesophageal echocardiography. Am J Cardiol. (2010) 106:885–92. 10.1016/j.amjcard.2010.05.014 20816132

[106] Morcos R Al Taii H Bansal P Casale J Manam R Patel V. Accuracy of commonly-used imaging modalities in assessing left atrial appendage for interventional closure: review article. J Clin Med. (2018) 7:13. 10.3390/jcm7110441 30441824 PMC6262547

[107] Holmes DR Reddy VY Turi ZG Doshi SK Sievert H Buchbinder M. Investigators, percutaneous closure of the left atrial appendage vs. warfarin therapy for prevention of stroke in patients with atrial fibrillation: a randomised non-inferiority trial. Lancet. (2009) 374:534–42. 10.1016/S0140-6736(09)61343-X 19683639

[108] Yu SD Zhang HP Li HW. Cardiac computed tomography vs. transesophageal echocardiography for the detection of left atrial appendage thrombus: a systemic review and meta-analysis. J Am Heart Assoc. (2021) 10:23. 10.1161/JAHA.121.022505 34796743 PMC9075398

[109] Morelos-Guzman M Minero-Garcia L Jaramillo-Almaguer JE Chavez-Carbajal JF Arean-Martinez CA Vargas-Espinosa JM. A novel, non-invasive, integral imaging assessment in atrial fibrillation by cardiac tomography. Arch Cardiol Mex. (2021) 91:41–8. 10.24875/ACME.M21000174 33008155 PMC8258911

[110] Vira T Pechlivanoglou P Connelly K Wijeysundera HC Roifman I. Cardiac computed tomography and magnetic resonance imaging vs. transoesophageal echocardiography for diagnosing left atrial appendage thrombi. Europace. (2019) 21:E1–E10. 10.1093/europace/euy142 29961869

[111] Muellerleile K Sultan A Groth M Steven D Hoffmann B Adam G. Velocity encoded cardiovascular magnetic resonance to assess left atrial appendage emptying. J Cardiov Magn Reson. (2012) 14:39. 10.1186/1532-429X-14-39 22720796 PMC3434119

[112] Camm AJ Lip GYH De Caterina R Savelieva I Atar D Hohnloser SH. 2012 focused update of the ESC guidelines for the management of atrial fibrillation an update of the 2010 ESC guidelines for the management of atrial fibrillation developed with the special contribution of the European Heart Rhythm Association. Europace. (2012) 14:1385–413. 10.1093/europace/eus305 22923145

[113] January CT Wann LS Calkins H Chen LY Cigarroa JE Cleveland JC. 2019 AHA/ACC/HRS Focused Update of the 2014 AHA/ACC/HRS guideline for the management of patients with atrial fibrillation a report of the American College of Cardiology/American Heart Association Task Force on Clinical Practice Guidelines and the Heart Rhythm Society. J Am Coll Cardiol. (2019) 74:104–32. 10.1016/j.jacc.2019.01.011 30703431

[114] Kirchhof P Benussi S Kotecha D Ahlsson A Atar D Casadei B. 2016 ESC Guidelines for the management of atrial fibrillation developed in collaboration with EACTS. Eur Heart J. (2016) 37:2893-962. 10.5603/KP.2016.0172 27567408

[115] Marsico F Cecere M Parente A Paolillo S de Martino F Dellegrottaglie S. Effects of novel oral anticoagulants on left atrial and left atrial appendage thrombi: an appraisal. J Thromb Thrombolysis. (2017) 43:139–48. 10.1007/s11239-016-1421-9 27614756

[116] Schnieder M Siddiqui T Karch A Bahr M Hasenfuss G Schroeter MR. Low flow in the left atrial appendage assessed by transesophageal echocardiography is associated with increased stroke severity-Results of a single-center cross-sectional study. Int J Stroke. (2019) 14:423–9. 10.1177/1747493018816511 30480476

[117] Gawalko M Budnik M Uzieblo-Zyczkowska B Krzesinski P Scislo P Kochanowski J. Decreased left atrial appendage emptying velocity as a link between atrial fibrillation type, heart failure and older age and the risk of left atrial thrombus in atrial fibrillation. Int J Clin Pract. (2020) 74:9. 10.1111/ijcp.13609 32654352

[118] Yaggi HK Concato J Kernan WN Lichtman JH Brass LM Mohsenin V. Obstructive sleep apnea as a risk factor for stroke and death. N Engl J Med. (2005) 353:2034–41. 10.1056/NEJMoa043104 16282178

[119] Pereg D Rozenbaum Z Vorobeichik D Shlomo N Gilad R Bloch S. Prevalence and significance of unrecognized renal dysfunction in patients with stroke. Am J Med. (2016) 129:1074–81. 10.1016/j.amjmed.2016.05.003 27215905

[120] Hayashi K Tsuda T Nomura A Fujino N Nohara A Sakata K. Impact of B-type natriuretic peptide level on risk stratification of thromboembolism and death in patients with nonvalvular atrial fibrillation - the hokuriku-plus AF registry. Circ J. (2018) 82:L1271-8. 10.1253/circj.CJ-17-1085 29491320

[121] Jin C Feng JJ Wang L Yu H Liu J Lu JW. Left atrial appendage segmentation using fully convolutional neural networks and modified three-dimensional conditional random fields. IEEE J Biomed Health Inf. (2018) 22:1906–16. 10.1109/JBHI.2018.2794552 29994758

[122] Jin C Feng JJ Wang L Yu H Liu J Lu JW. Left atrial appendage segmentation and quantitative assisted diagnosis of atrial fibrillation based on fusion of temporal-spatial information. Comput Biol Med. (2018) 96:52–68. 10.1016/j.compbiomed.2018.03.002 29547711

[123] Morais P Queiros S De Meester P Budts W Vilaca JL Tavares J. Fast segmentation of the left atrial appendage in 3-D transesophageal echocardiographic images. IEEE Trans Ultrason Ferroelectr Freq Control. (2018) 65:2332–42. 10.1109/TUFFC.2018.2872816 30281444

[124] Qiao MY Wang YY Berendsen FF van der Geest RJ Tao Q. Fully automated segmentation of the left atrium, pulmonary veins, and left atrial appendage from magnetic resonance angiography by joint-atlas-optimization. Med Phys. (2019) 46:2074–84. 10.1002/mp.13475 30861147 PMC6849806

[125] Leventic H Babin D Velicki L Devos D Galic I Zlokolica V. Left atrial appendage segmentation from 3D CCTA images for occluder placement procedure. Comput Biol Med. (2019) 104:163–74. 10.1016/j.compbiomed.2018.11.006 30481731

[126] Mill J Agudelo V Olivares AL Pons MI Silva E Nunez-Garcia M. Sensitivity analysis of in silico fluid simulations to predict thrombus formation after left atrial appendage occlusion. Mathematics. (2021) 9:1–14. 10.3390/math9182304

[127] Duenas-Pamplona J Sierra-Pallares J Garcia J Castro F Munoz-Paniagua J. Boundary-condition analysis of an idealized left atrium model. Ann Biomed Eng. (2021) 49:1507–20. 10.1007/s10439-020-02702-x 33403454

